# Isolated right ventricular noncompaction caused ventricular tachycardia and pulmonary embolism

**DOI:** 10.1111/anec.12731

**Published:** 2019-11-20

**Authors:** Qian Huo, Liwen Liang, Xiaoxue Ding, Mingjie Pang, Yan Zhao, Hong Zhang, Wenhua Su

**Affiliations:** ^1^ Department of Geriatrics First People's Hospital of Yunnan Province Kunming China; ^2^ Department of Cardiology First People's Hospital of Yunnan Province Kunming China; ^3^ Department of Medicine Kunming University of Science and Technology Kunming China

**Keywords:** isolated right ventricular noncompaction, pulmonary embolism, ventricular tachycardia

## Abstract

Isolated ventricular noncompaction is an unclassified cardiomyopathy due to intrauterine arrest of compaction of the loose interwoven meshwork. Its mortality and morbidity are high, including heart failure, thromboembolic events, and ventricular arrhythmias. Isolated right ventricular noncompaction was reported rarely, especially that causes pulmonary embolism and ventricular tachycardia. We describe a case of isolated noncompaction of the right ventricular causing pulmonary embolism and ventricular tachycardia.

## 1 INTRODUCTION

Ventricular noncompaction (VNC) is a rare congenital heart disease that results from an abnormal arrest in endomyocardial embryogenesis. It is characterized by the presence of prominent ventricular myocardial trabeculations and deep intratrabecular crypts, which normally ventricular myocardial trabeculations usually involves in the left ventricular(LV) apex while rare in the bi‐ventricles or right ventricle (RV) (Weiford, Subbarao, & Mulhern, 2004).We present a case of isolated right ventricular noncompaction (iRVNC) presenting as ventricular tachycardia (VT) and pulmonary embolism (PE).

## 2 CASE REPORT

**Figure 1:**
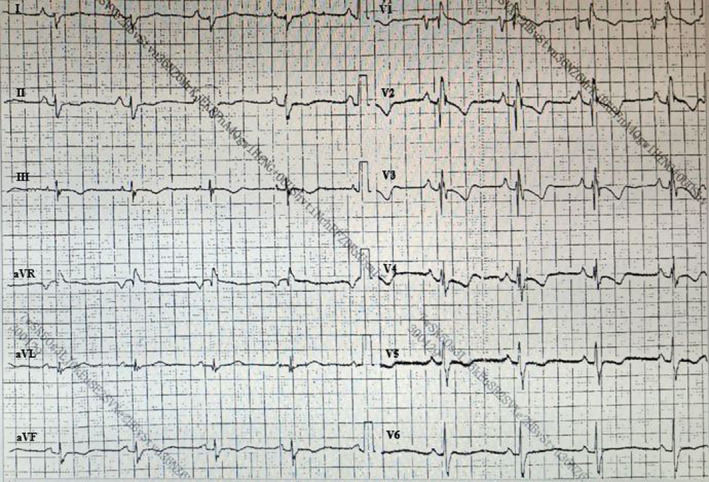
The twelve‐lead electrocardiogram showed sinus rhythm with right bundle branch block

**Figure 2:**
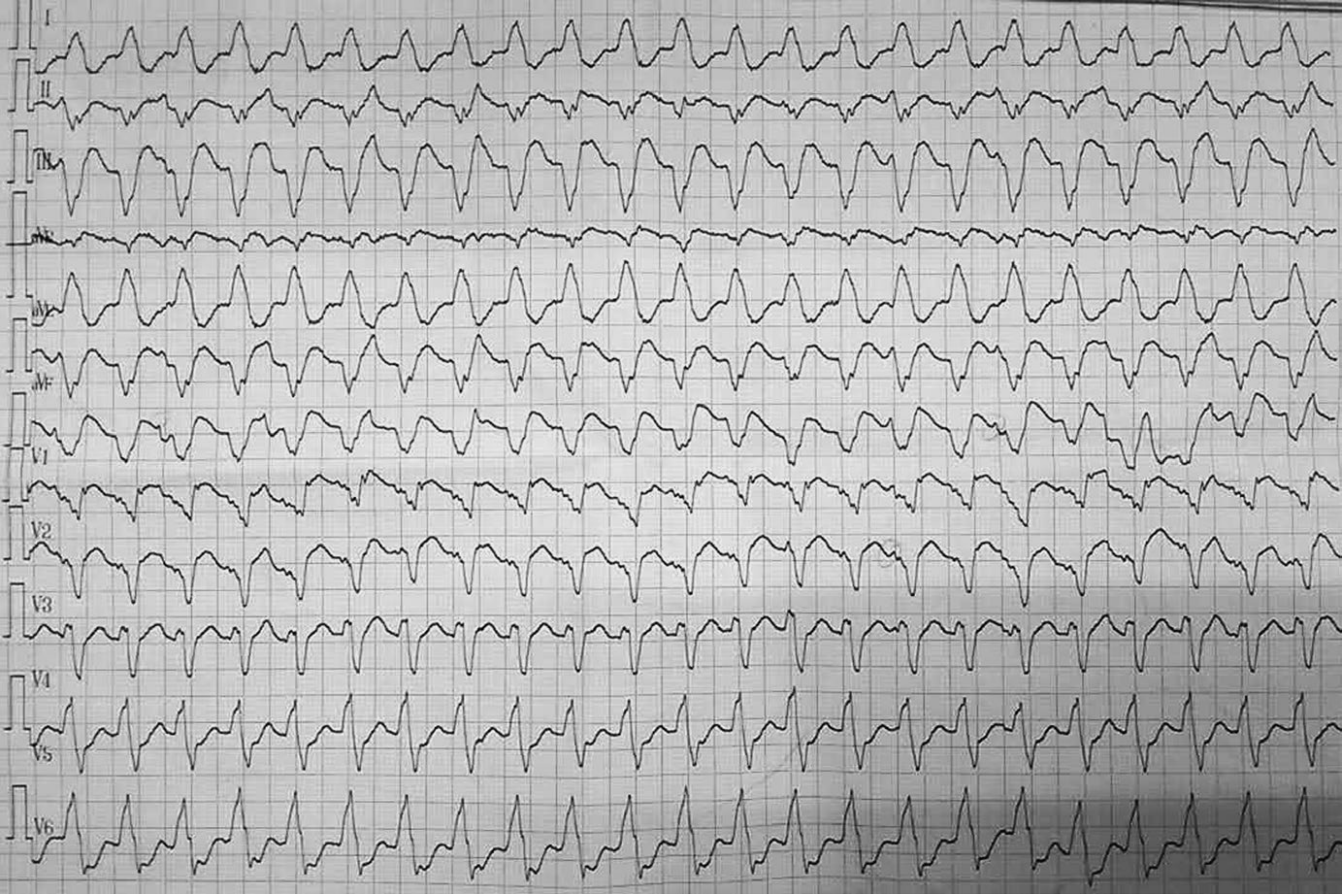
Holter monitoring revealed ventricular tachycardia at a rate of 150/min with left bundle branch block morphology

**Figure 3:**
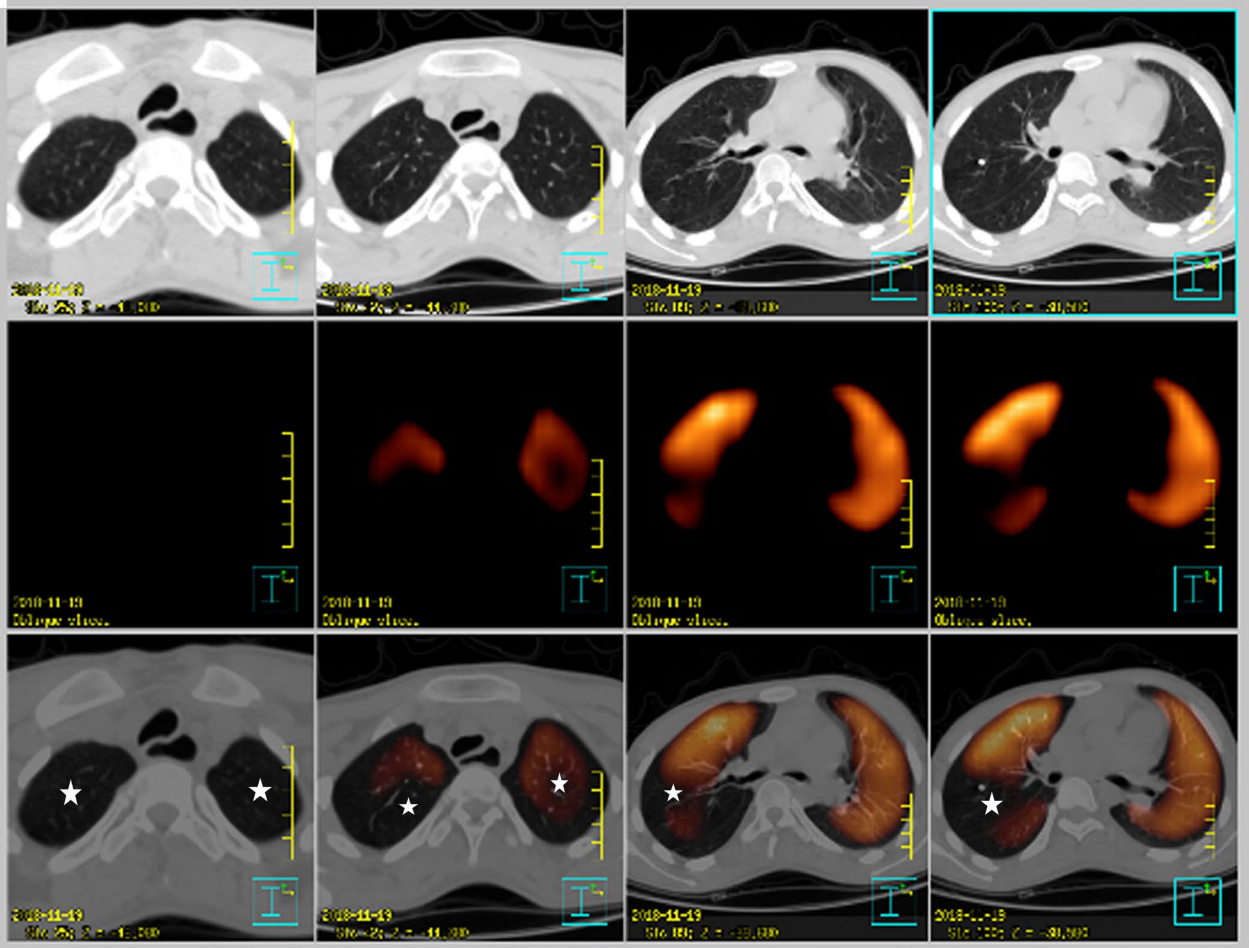
Several perfusion defects (asterisks) on dorsal segment in the apicoposterius segment of the upper lobe of the bi‐lung and the dorsal segment of the lower lobe of the right lung

**Figure 4:**
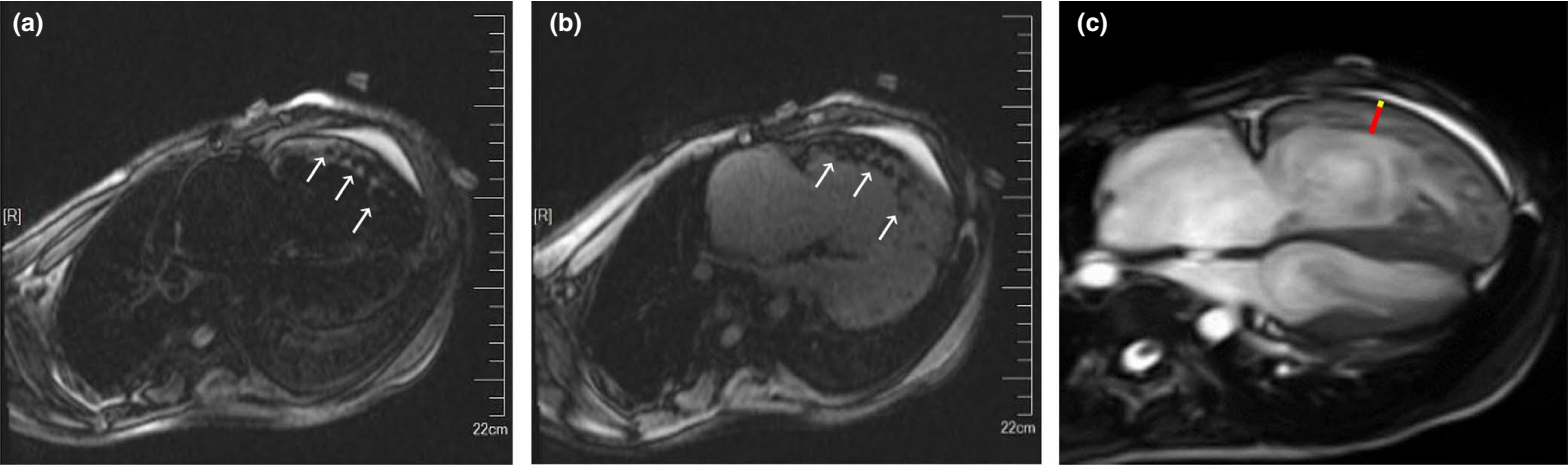
Panel (a) and (b) show the prominent trabeculations and deep intertrabecular recesses (arrows) of the right ventricular wall. Panel (c) demonstrates long‐axis noncompaction ratio measurement (red line identifies the end‐diastolic thickness of the noncompacted layer, yellow line identifies the end‐diastolic thickness of the compacted layer) with a maximum long‐axis noncompaction ratio of 4.17 obtained in the right ventricular wall

A 20‐year‐old man presented with repeated amaurosis and progressive dyspnea for the past 6 months. He was never found to have any heart diseases, relevant history of familial heart diseases, and risk factors for pulmonary embolism in the past. On examination, his blood pressure was 112/68 mmHg, pulse 104 beats per minute. The lips appeared cyanosis, and the jugular vein was filling together with negative liver‐jugular vein backflow sign (−). His bi‐lung breath sounded clear, and the heartbeat sounded strong with regular heart rhythm, P2 > A2; valve auscultation did not appear any noise, and the both lower extremities showed mild edema. D‐dimer 2.80 g/ml (0–0.3 g/ml), blood gas analysis: arterial oxygen partial pressure 66 mmHg (75–100 mmHg), arterial carbon dioxide partial pressure 38.6 mmHg (35–45 mmHg). Brain natriuretic peptide 2843 pg/ml (0–100 pg/ml). The twelve‐lead electrocardiogram showed sinus rhythm with right bundle branch block (Figure 1). Holter monitoring revealed ventricular tachycardia at a rate of 150/min with left bundle branch block morphology (Figure 2). Transthoracic echocardiogram revealed the right ventricular and atrium was remarkably dilated, anterior myocardium of right ventricular became thinner and hypokinetic. Pulmonary artery pressure increased considerably (72 mmHg). The left ventricular was not markedly dilated, and its function was normal with a left ventricular ejection fraction of 55%. Single‐photon emission computed tomography (SPECT) imaging (Figure 3) showed pulmonary embolism, which was found in the apicoposterius segment of the upper lobe of the bi‐lung and the dorsal segment of the lower lobe of the right lung. There was no abnormality in lower extremity B‐ultrasound. Cardiac magnetic resonance imaging (MRI) (Figure 4) confirmed the diagnosis of iRVNC and no evidence of fatty infiltration. The patient's whole exon sequencing did not find anything meaningful.

In the electrophysiology laboratory, rapid ventricular pacing induced the clinical VT. Implantable cardioverter‐defibrillator was recommended, while the patient and his parents refused resolutely. Electroanatomic mapping and pace mapping located the VT origin to the right ventricular inferior wall. Radiofrequency ablation induced VT at this site; after several radiofrequency ablation applications, VT was no longer inducible. Besides, the patient was treated with heparin sodium and warfarin (anticoagulation), angiotensin‐converting enzyme inhibitors (ACEI), β‐receptor inhibitors, and spironolactone so as to improve the cardiac function. The 8‐month follow‐up revealed no recurrence of amaurosis and dyspnea chest tightness, no further episodes of VT occurred, and still needs long‐term follow‐up.

## 3 DISCUSSION

The pathogenesis of VNC is not clear yet. Normally between the fetal 5th and 8th week, intertrabecular spaces are obliterated and ventricular compaction occurs from the base toward the apex and from epicardium to endocardium. VNC is generally thought to be caused by arrest of normal embryogenesis of the endocardium and myocardium (Weiford et al., 2004). Isolated left ventricular noncompaction (iLVNC) was proposed four diagnostic criteria (Jenni, Wyss, & Oechslin, 2002), which have been widely used, while, due to different anatomical characteristics, iRVNC is more difficultly diagnosed in clinics than iLVNC. Electrocardiograph, echocardiogram, computed tomography, and MRI are recommended as the diagnostic models (Weiford et al., 2004).

Major morbidity during long‐term follow‐up included heart failure, arrhythmias, and thromboembolic events. Heart failure, caused by systolic and diastolic dysfunction, was the most common cause for hospital admission. Cases of noncompaction isolated to the RV without LV involvement have been reported; however, they are often incidentally found and have not been described to cause PE and VT. We describe a case of isolated noncompaction of the RV causing PE and VT.

The electrophysiological mechanism of arrhythmia is still unclear, which may be linked to the irregular connection of the trabeculae and branches of the myocardium, and the instability of myocardial electrophysiology caused by insufficient blood supply to the myocardium. The VT of the patient in this report was successfully eliminated with catheter ablation.

Clinically, PE mostly comes from the lower extremity deep vein system, but if such patients have no evidence of deep venous thrombosis, other sources of emboli should be considered. It is very rare that the embolus comes from the right heart system (in situ thrombosis). The patient reported in this study was diagnosed as PE by SPECT. Since the patient was never found to have any risk factors for PE in the past and no thrombus was found in ultrasound in both lower limb varices, it was thought the PE was caused by situ embolus of the RV. The deep intertrabecular space may aggravate the risk of thrombus formation and be an additional factor for the situ embolus of the RV. The patients with PE, especially those without significant risk factors or with normal results in lower extremity deep venous system examination, should be considered the possibility of this disease. One follow‐up toward 34 adults with VNC recommended anticoagulants should be administered independent of ventricular function to prevent embolic complications (Oechslin, Attenhofer Jost, & Rojas, 2000). The patient in this report was treated with long‐term warfarin anticoagulation therapy together with oral ACEI, β‐block inhibitors, and spironolactone simultaneously so as to improve the right heart function.

## 4 CONCLUSION

Isolated ventricular noncompaction is a primary cardiomyopathy that affects the left ventricle and is known to cause arrhythmias and thromboembolic events. Case of on‐compaction isolated to the right ventricular without left ventricular involvement has not been described to cause VT and PE. We describe a case of iRVNC causing VT and PE successfully treated with catheter ablation and anticoagulation separately. iRVNC is very rare in clinics, and can lead to PE and VT, so serious attention should be given clinically.

## CONFLICT OF INTEREST

None of the authors have any conflict of interest to disclose.

## ETHICAL APPROVAL AND CONSENT TO PARTICIPATE

This study was approved by the *First People's Hospital of Yunnan Province* and informed consent was obtained from the patient.
